# Molecular Recognition and Quantification of HER-3, HER-4 and HRG-α in Whole Blood and Tissue Samples Using Stochastic Sensors

**DOI:** 10.3390/mi13101749

**Published:** 2022-10-15

**Authors:** Raluca-Ioana Stefan-van Staden, Damaris-Cristina Gheorghe

**Affiliations:** 1 Laboratory of Electrochemistry and PATLAB, National Institute of Research for Electrochemistry and Condensed Matter, 202 Splaiul Independentei Str., 060021 Bucharest, Romania; 2 Faculty of Chemical Engineering and Biotechnologies, University Politehnica of Bucharest, 011061 Bucharest, Romania

**Keywords:** stochastic sensors, human epidermal growth factor receptor-3, human epidermal growth factor-receptor-4, heregulin-α

## Abstract

Human epidermal growth factor receptor-3, human epidermal growth factor-receptor-4, and heregulin-α are some of the biomarkers related to gastric cancer currently being used for early detection, personalized treatment, and evaluation of the efficiency of the treatment. Two stochastic sensors based on graphene decorated with TiO_2_ and/or Au modified with maltodextrin were proposed for the screening of two types of whole blood and tissue samples for the simultaneous recognition and analysis of the three biomarkers. The sensitivity of the two sensors showed high values, whereas the limits of determination were of fg mL^−1^ magnitude order. Thus, the proposed screening method can perform the quantitative analysis of both of the biomarkers of interest in whole blood and tissue samples, with recoveries higher than 96.00% and relative standard deviations lower than 1.00%.

## 1. Introduction

Heregulin-α (HRG-α) is known to be involved in both pathological and physiological processes of epithelial cells from various types of organs [1]. Human epidermal growth factor receptor-3 (HER-3) and human epidermal growth factor-receptor-4 (HER-4) belong to the Her/ErbB family and are known for their involvement in a series of pathways which are related to angiogenesis and cell migration [2]. HER-3 is present in various types of tumors due to its somatic mutations [3]. HER-4 is the only type of receptor with growth-inhibiting properties [4]. Compared to the expression of HER-3, which is present in the final stages of various types of cancer, HER-4 is downregulated [5]. Therefore, their simultaneous assay in whole blood and tissue samples is of high importance.

Throughout the years, stochastic sensors have been utilized for the purpose of analyzing a variety of biomarkers from biological samples in order to diagnose diseases such as diabetes [6] and lung cancer [7]. Multiple matrices, including graphene, carbon nanotubes, single- and multi-wall carbon nanotubes, graphite powder, and diamond paste, as well as modifiers, including α-cyclodextrin, 2,3,7,8,12,13,17,18-octaethyl-21H,23H-porphine manganese (III) chloride, and chitosan, were used to design these types of sensors.

Two stochastic sensors were proposed in this paper for molecular recognition and quantification of HER-3, HER-4, and HRG- in whole blood and tissue samples. The stochastic sensors were based on graphene decorated with TiO_2_ and/or Au modified with maltodextrin. Stochastic sensors [8,9,10,11] have many advantages; they are capable of assaying samples both qualitatively and quantitatively; they can perform a reliable quantitative analysis, no matter the complexity of the matrix; they are easy to use; and they are cost-effective. Graphene materials ensure a stable environment for the channels of maltodextrin needed for stochastic sensors [12]. Furthermore, the addition of TiO_2_ and/or Au improved the conductivity property of the graphene [12].

## 2. Materials and Methods

### 2.1. Chemicals

HRG-α, HER-3, HER-4, maltodextrin (MD), monosodium phosphate and disodium phosphate were purchased from Sigma Aldrich (Milwaukee, WI, USA); the paraffin oil was purchased from Fluka (Buchs, Switzerland). Monosodium phosphate and disodium phosphate were used for the preparation of phosphate buffer (PBS), pH = 7.40. The obtained PBS was used for the preparation of the HRG-α, HER-3 and HER-4 solutions.

The standard solutions had the following concentrations: HRG-α 4.09 × 10^−9^ to 5.00 μg mL^−1^, HER-3 and HER-4 1.00 to 1.00 × 10^−9^ μg mL^−1^; these solutions were diluted using the serial dilution method up to fg mL^−1^ magnitude order. The solutions were used for one month; when not in use, they were kept at a temperature of 2–8 °C.

### 2.2. Instruments

In order to record all measurements, an EmStatpico (Houten, The Netherlands) connected to a personal computer with a PSTrace (Version 5.9) software was used. An electrochemical cell, containing a three-electrode system, was also employed. The three-electrode system is made out of: the counter electrode, represented by a platinum wire; the working electrode, which in our case is represented by the two proposed stochastic sensors; and the reference electrode, which is also known as the Ag/AgCl electrode.

In order to determine the pH of the solutions, a pH meter (Mettler Toledo Seven Compact pH meter S210) was employed.

### 2.3. Sensors Design

**Scheme 1:**
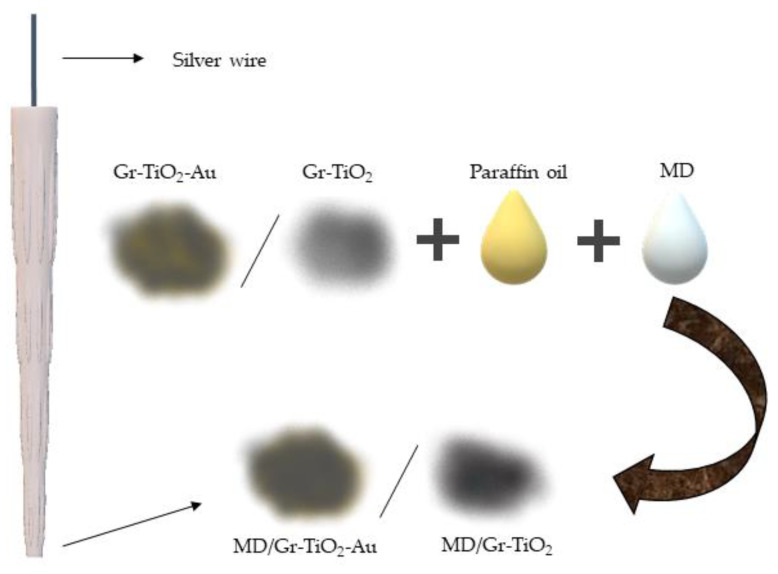
Schematic representation of the design of the stochastic sensors.

Two types of graphene decorated with TiO_2_ and with TiO_2_-Au were mixed with paraffin oil, until two homogeneous pastes were obtained. Solutions of 100 μL of maltodextrin (10^−3^ mol L^−1^), were added to 100 mg of each of the graphene pastes to obtain the modified paste for the stochastic sensors: MD/Gr-TiO_2_ and MD/Gr-TiO_2_-Au (Scheme 1). Each modified paste was placed in a non-conducting plastic tube, 3D printed in the laboratory, presenting an internal diameter of 100 μm. In order to connect the pastes and the external circuit, a silver wire was used. Before and after each measurement, the sensors were cleaned with distilled water. When not in use, the proposed stochastic sensors were kept at room temperature.

### 2.4. Stochastic Mode

**Figure 1:**
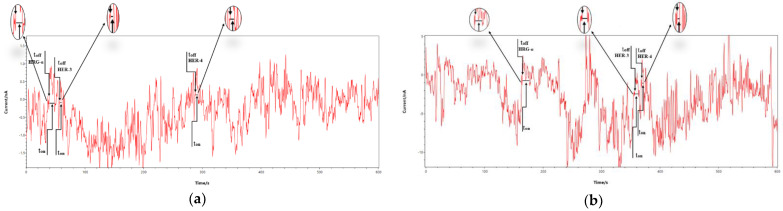
Pattern recognition of HRG−α, HER−3 and HER−4 in whole blood samples, using stochastic sensors: (**a**) MD/Gr−TiO_2_, (**b**) MD/Gr−TiO_2_−Au.

**Figure 2:**
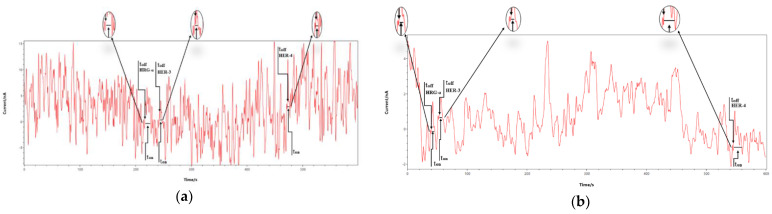
Pattern recognition of HRG−α, HER−3 and HER−4 in tissue samples using stochastic sensors: (**a**) MD/Gr−TiO_2_, (**b**) MD/Gr−TiO_2_−Au.

All measurements presented in this paper were obtained when a chronoamperometric method was employed, at a constant potential of 180 mV. The principle of the stochastic sensors is based on the channel conductivity, when a constant potential is applied and the current is recorded [9]. For these sensors, the optimization of the current was necessary in order to find the right potential that offers the desired response, and 180 mV was used for all of these measurements. No stochastic response was obtained when other potentials (between 0 and 179 mV and between 181 and 500 mV) were applied. The parameters of interest are t_off_ and t_on_. Using the t_off_ values, which are specific for each biomarker, each analyte was identified on the recorded diagrams (Figure 1 and Figure 2). Calibration equations were obtained using the linear regression method; for each equation of calibration, 12 points were considered. The unknown concentrations for HRG-α, HER-3 and HER-4 were determined using the t_on_ values, which were identified in the obtained diagrams.

When a constant potential of 180 mV is applied, the current flowing through a channel is modified when the analytes of interest bind with the wall channel. In order for the analytes of interest to be molecularly recognised, two phases need to be completed. The first phase is known as the molecular recognition phase; the analytes of interest are extracted from the solution into the membrane-solution of the interface and block the channel; because of this, the intensity of the channel decreases to around 0 for a certain period of time, which is known as t_off_ and which represents the signature of the biomarkers; its value is used for the qualitative analysis of the analytes of interest and is unique for each biomarker.

The second phase Is known as the binding phase and takes place when the analytes interact with the wall channel. During the second phase, the following equations of equilibrium are obtained:

Ch(i) + HRG-α(i) ↔ Ch • HRG-α(i)

Ch(i) + HER-3(i) ↔ Ch • HER-3(i)

Ch(i) + HER-4(i) ↔ Ch • HER-4(i)

where Ch represents the channel and i represents the interface. The quantitative parameter, also known as t_on_, represents the equilibrium time necessary for the interaction between the analytes and the wall channel. The t_on_ values are determined in accordance with the following equation 1/t_on_ = a + b × C_HRG-α/HER-3/HER-4_ (Table 1).

### 2.5. Samples

For this study, five whole blood and five tissue samples were received from the County Emergency Hospital from Targu-Mures. These samples belonged to confirmed patients with gastric cancer and were collected according to the procedures specified by the Ethics committee, approval number 32647/2018, awarded by the County Emergency Hospital from Targu-Mures. When not in use, both types of samples were kept at a temperature of −80 °C. Written consent was obtained from all patients. Moreover, no sample pre-treatment was performed.

## 3. Results and Discussion

### 3.1. Response Characteristics of Stochastic Osensors

Chronoamperometry made possible the use of a constant potential of 180 mV, which was applied for all measurements. The biomarkers of interest enter the channel of the used electrodes when the mentioned potential is applied; at this stage, the intensity of the current drops to zero (the necessary time to enter and exit the channel is known as the signature of the analyte and it is marked on the diagrams as t_off_). As long as the biomarkers stay inside the channel, they undergo both redox and binding processes—the time required for these is marked as t_on_.

The response characteristics of the proposed stochastic sensors are shown in Table 1. The results are the average of ten determinations. The molecular recognition of the biomarkers of interest from the assayed biological samples was made possible by their different t_off_ values.

The widest linear concentration ranges were obtained for the sensor based on MD/Gr-TiO_2_-Au. The best sensitivities were: for the assay of HER-3 6.03 × 10^10^ s^−1^/g mL^−1^ and for the assay of HRG-α 2.33 × 10^12^ s^−1^/g mL^−1^, when the sensor based on MD/Gr-TiO_2_-Au, while for the assay of HER-4 the best sensitivity (6.43 × 10^11^ s^−1^/g mL^−1^) was obtained using the sensor based on MD/Gr-TiO_2_. The lowest limits of quantification for all three biomarkers were obtained when the sensor based on MD/Gr-TiO_2_-Au was used.

### 3.2. The Reliability and Stability of the Stochastic Sensors

Ten stochastic sensors from each of the two types were designed, and the sensitivity was determined for each of them. These determinations performed for each type of microsensor proved that there are no significative changes in the sensitivity, its variation being for each type of microsensor lower than 0.08%; this proved the reproducibility of the design of each type of stochastic sensor. The stochastic sensors were tested for a period of one year, to check their stability in time. During this period of time, their sensitivity varied, with 1.2% for the sensor based on MD/Gr-TiO_2_-Au and 1.5% for the sensor based on MD/Gr-TiO_2_, proving good stability in time and that the proposed design is also reliable.

### 3.3. Selectivity of the Sensors

The selectivity of the stochastic sensors is given by the difference between the signatures (t_off_ values) recorded for HER-3, HER-4 and HRG-α and those obtained for other biomarkers/substances from the biological samples. The possible interfering species selected were: p53, maspin and CA19-9. The values obtained for the signatures proved the selectivity of the proposed sensors (Table 2).

### 3.4. Determination of HER-3, HER-4 and HRG-α in Whole Blood and Tissue Samples

Five whole blood and five tissue samples were screened according to the method previously described. By using the specific signatures of each biomarker, they were identified on the diagrams, which can be found in Figure 1 and Figure 2. The most important step is represented by the identification of the signature of the biomarkers; after this, the t_on_ values were also identified. Very good correlations between the results were obtained when both sensors were used. The results presented in Table 3 and Table 4 proved that HER-3, HER-4 and HRG-α can be determined from biological samples (whole blood and tissue) using the proposed stochastic sensors.

For further validation, real samples of whole blood and tissue were spiked with known amounts of the three biomarkers, and recovery tests were performed. The results are shown in Table 5.

The results shown in Table 5 proved that the proposed stochastic sensors can be used reliably for the molecular recognition and for the quantification of HER-3, HER-4 and HRG-α in whole blood and tumor tissue samples.

## 4. Conclusions

The purpose of this paper was to develop a reliable screening test for molecular recognition of three gastric cancer biomarkers (HER-3, HER-4 and HRG-α) from biological samples. Two stochastic sensors were used in order to perform the analysis of the samples and the obtained results showed high sensitivity, low limits of determination and high reliability. This method is for implementation in clinical study for better understanding of the correlation between HER-3, HER-4, with HRG-α quantities, in order to facilitate the early detection of gastric cancer, to adopt a personalized treatment, and to determine as soon as possible if the treatment given to the patient is efficient or not.

## Figures and Tables

**Table 1:** The response characteristics of stochastic sensors used for the assay of HER-3, HER-4 and HRG-α.

Stochastic Microsensor Modified with MD and Based on	t_off_ (s)	Equation of Calibration *, r	Sensitivity (s^−1^/g mL^−1^)	LOQ (g mL^−1^)	Linear Concentration Range (g mL^−1^)	LOD (g mL^−1^)
**Gr-TiO_2_-Au**	**HER-3**					
	2.2	1/t_on_ = 0.11(±0.02) + 6.03(±0.04) × 10^10^ C r = 0.9999	6.03(±0.04) × 10^10^	10^−15^	10^−15^–10^−11^	3.7 × 10^−16^
	**HER-4**					
	0.8	1/t_on_ = 0.09(±0.01) + 4.94(±0.03) × 10^10^ C r = 0.9992	4.94(±0.03) × 10^10^	10^−15^	10^−15^–10^−6^	2.0 × 10^−16^
	**HRG-α**					
	1.4	1/t_on_ = 0.05(±0.01) + 2.33(±0.02) × 10^12^ C r = 0.9998	2.33(±0.02) × 10^12^	2.05 × 10^−14^	2.05 × 10^−14^–5 × 10^−6^	2.0 × 10^−14^
**Gr-TiO_2_**	**HER-3**					
	1.1	1/t_on_ = 0.22(±0.03) + 2.32(±0.02) × 10^9^ C r = 0.9992	2.32(±0.02) × 10^9^	10^−14^	10^−14^–10^−10^	2.8 × 10^−15^
	**HER-4**					
	1.4	1/t_on_ = 0.27(±0.03) + 6.43(±0.02) × 10^11^ C r = 0.9997	6.43(±0.02) × 10^11^	10^−14^	10^−14^–10^−10^	3.7 × 10^−15^
	**HRG-α**					
	1.7	1/t_on_ = 0.28(±0.02) + 1.69(±0.03) × 10^10^ C r = 0.9996	1.69(±0.03) × 10^10^	5.12 × 10^−13^	5.12 × 10^−13^–1.28 × 10^−11^	1.2 × 10^−14^

* <C> = g mL^−1^ <t_on_> = s.

**Table 2:** Selectivity of the stochastic sensors.

Stochastic Microsensor Modified with MD and Based on	Signatures (t_off_ Values), s					
	HER-3	HER-4	HRG-α	p53	Maspin	CA19-9
**Gr-TiO_2_-Au**	2.2	0.8	1.4	3.4	2.7	0.3
**Gr-TiO_2_**	1.1	1.4	1.7	3.2	2.5	0.5

**Table 3:** Determination of HER-3, HER-4 and HRG-α in whole blood samples.

Sample No.	Biomarker	Stochastic Microsensor Based on MD/Gr-TiO_2_-Au	Stochastic Microsensor Based on MD/Gr-TiO_2_	ELISA
		pg mL^−1^		
1	HER-3	385.61 ± 0.08	383.23 ± 0.10	385.00
	HER-4	7.75 ± 0.14	7.83 ± 0.12	7.78
	HRG-α	32.45 ± 0.12	32.51 ± 0.11	32.50
2	HER-3	156.09 ± 0.10	157.80 ± 0.12	157.00
	HER-4	8.23 ± 0.03	8.95 ± 0.08	8.20
	HRG-α	28.34 ± 0.09	28.73 ± 0.10	28.30
3	HER-3	268.81 ± 0.15	263.34 ± 0.14	264.00
	HER-4	136.86 ± 0.10	139.80 ± 0.12	135.95
	HRG-α	57.14 ± 0.08	57.11 ± 0.09	57.00
4	HER-3	91.47 ± 0.13	91.33 ± 0.18	91.20
	HER-4	0.49 ± 0.07	0.45 ± 0.05	0.40
	HRG-α	4.11 ± 0.03	4.39 ± 0.08	4.10
5	HER-3	148.50 ± 0.18	143.45 ± 0.12	145.40
	HER-4	0.68 ± 0.05	0.70 ± 0.04	0.60
	HRG-α	1.63 ± 0.12	1.76 ± 0.21	1.62

**Table 4:** Determination of HER-3, HER-4 and HRG-α in tissue samples.

Sample No.	Biomarker	Stochastic Microsensor Based on MD/Gr-TiO_2_-Au	Stochastic Microsensor Based on MD/Gr-TiO_2_	ELISA
		pg mL^−1^		
1	HER-3	211.73 ± 0.21	211.96 ± 0.14	211.12
	HER-4	5.34 ± 0.14	5.43 ± 0.12	5.30
	HRG-α	27.09 ± 0.12	25.48 ± 0.14	26.03
2	HER-3	685.21 ± 0.11	684.18 ± 0.12	684.00
	HER-4	6.91 ± 0.08	7.34 ± 0.10	6.95
	HRG-α	16.35 ± 0.05	16.11 ± 0.08	16.12
3	HER-3	6.85 ± 0.12	6.43 ± 0.13	6.80
	HER-4	4.44 ± 0.10	4.26 ± 0.08	4.40
	HRG-α	8.03 ± 0.12	8.98 ± 0.15	8.00
4	HER-3	147.52 ± 0.11	143.45 ± 0.10	146.00
	HER-4	18.36 ± 0.12	18.98 ± 0.09	18.80
	HRG-α	27.50 ± 0.11	25.48 ± 0.13	26.93
5	HER-3	56.86 ± 0.10	57.82 ± 0.13	56.95
	HER-4	6.91 ± 0.05	6.98 ± 0.08	6.90
	HRG-α	7.55 ± 0.03	7.91 ± 0.04	7.63

**Table 5:** Recovery of HER-3, HER-4 and HRG-α in whole blood and tumoral tissue samples.

Stochastic Microsensor Modified with MD and Based on	Biological Sample	%, Recovery		
		HER-3	HER-4	HRG-α
**Gr-TiO_2_-Au**	Whole blood	98.78 ± 0.02	99.03 ± 0.01	99.16 ± 0.03
**Gr-TiO_2_**	Tumor tissue	96.98 ± 0.03	97.95 ± 0.05	98.04 ± 0.05

## Data Availability

Not applicable.
